# Depicting the mating system and patterns of contemporary pollen flow in trees of the genus *Anadenanthera* (Fabaceae)

**DOI:** 10.7717/peerj.10579

**Published:** 2021-04-07

**Authors:** Juliana Massimino Feres, Alison G. Nazareno, Leonardo M. Borges, Marcela Corbo Guidugli, Fernando Bonifacio-Anacleto, Ana Lilia Alzate-Marin

**Affiliations:** 1 Programa de Pós-Graduação em Genética, Departamento de Genética, Universidade de São Paulo, Faculdade de Medicina de Ribeirão Preto, Ribeirão Preto, SP, Brazil; 2 Departamento de Genética, Ecologia e Evolução, Universidade Federal de Minas Gerais, Belo Horizonte, Minas Gerais, Brazil; 3 Departamento de Botânica, Universidade Federal de São Carlos, São Carlos, SP, Brazil

**Keywords:** Atlantic forest, Biparental inbreeding, Categorical paternity, Conservation genetics, Microsatellite markers, Gene flow

## Abstract

*Anadenanthera* (Fabaceae) is endemic to the Neotropics and consists of two tree species: *A. colubrina* (Vell.) Brenan and *A. peregrina* (L.) Speg. This study examined the mating system and contemporary gene flow of *A. colubrina* (Acol) and *A. peregrina* (Aper) in a highly fragmented area of the Atlantic Forest to provide valuable information that informs conservation strategies. Reproductive adults from forest remnants [n*_A. colubrina_* = 30 (2.7 ha), n*_A. peregrina_* = 55 (4.0 ha)] and progeny-arrays (n*_A. colubrina_* = 322, n*_A. peregrina_* = 300) were genotyped for seven nuclear microsatellite markers. Mating system analyses revealed that *A. colubrina* is a mixed mating species (*t_m_* = 0.619) while *A. peregrina* is a predominantly outcrossing species (*t_m_* = 0.905). For both *Anadenanthera* species, high indices of biparental inbreeding were observed (Acol = 0.159, Aper = 0.216), resulting in low effective pollination neighborhood sizes. Categorical paternity analysis revealed different scales of pollen dispersal distance: the majority of crossings occurring locally (i.e., between nearby trees within the same population), with moderate pollen dispersal coming from outside the forest fragments boundaries (Acol*_mp_* = 30%, Aper*_mp_* = 35%). Nevertheless, pollen immigration from trees outside the populations for both species suggests that the populations are not reproductively isolated. This study highlights the importance of evaluating both mating system and contemporary gene flow for a better understanding of the biology of *Anadenanthera* species. This information should be considered to ensure the effective conservation and management practices of these plant species.

## Introduction

Within in the semi-deciduous Brazilian Atlantic Forest, Ribeirão Preto is a region that has been significantly affected by land conversion for sugarcane agricultural practices. The municipality is currently among the largest producers of sugarcane in Brazil. Floristic survey data in this region indicate that Atlantic Forest remnants in Ribeirão Preto are distributed across 102 fragments (ranging from 1.5 to 247.0 ha), of which 54% are less than 10 ha (Kotchetkoff-Henriques, 2003). Among these fragments, 37 areas show a high density (more than 20 individuals/ha) of *Anadenanthera*, the target genus of this study. Plants of this genus often occur in monospecific clusters, known locally in Brazil as *angicais*. This population structure may be related to allelopathic effects that have been discussed in previous analyses of this genus (Silva et al., 2010). The regional landscape is marked by high concentrations of *Anadenanthera* scattered along highways and in municipal parks, especially in deciduous forest areas that are not suitable for agriculture, thus highlighting the importance of this genus in the region.

Occurring in Latin America and the Caribbean, *Anadenanthera* consists of two species and four varieties (Altschul, 1964). *Anadenanthera colubrina* (Vell.) Brenan includes two varieties: *A. colubrina* var. *colubrina*, which grows in northeastern Argentina and southeastern Brazil, and *A. colubrina* var. *cebil* (Griseb.) Altschul, which occurs in northern Argentina, Bolivia, Brazil, Paraguay, and Peru (Altschul, 1964). *Anadenanthera peregrina* (L.) Speg. consists of *A. peregrina* var. *peregrina*, which occurs in the northwest of Brazil, Guyana, Colombia, Venezuela, and the Antilles, and *A. peregrina* var. *falcata* (Benth.) Altschul which is found in southern Brazil and Paraguay. An in-depth botanic description of these species and their varieties, including detailed information on cultural and pharmacological uses can be found in Torres & Repke (2012).

Both species of the genus can be used in landscaping, and their excellent quality wood can be used in construction and shipbuilding (Lorenzi, 2002; Carvalho, 2003). The bark and the wood have tannin (used in traditional medicine) and a colorant that can be extracted from the bark (used in tanneries) (Lorenzi, 2002; Monteiro et al., 2006). *Anadenanthera* nuts contain alkaloids that were used by indigenous peoples in pre-colonial times, principally in Peru, Chile, Bolivia, and Argentina, and are thought to have potential medicinal properties (Weber et al., 2011; Torres & Repke, 2012). *Anadenanthera* species are considered honey plants as they provide bees with pollen and nectar (Souza et al., 2007). These species are widely recommended for the recovery of degraded and eroded areas, for riparian replacement in poor, shallow soils, and as a component of Agroforestry systems (Durigan & Silveira, 1999; Monteiro et al., 2006).

As the reduction of population size becomes more common due to ongoing loss and degradation of habitats in the Brazilian Atlantic Forest, it is essential to assess the mating parameters since they affect the genetic diversity of future generations and thus demonstrate a species’ responsiveness to anthropogenic factors. Over generations, mating parameters (e.g., effective dispersal distances of gene flow, and outcrossing rates) determine how genes are recombined and maintained in plant populations, providing the basis for the continuation of the evolutionary processes (e.g., Feres et al., 2009, 2012a; Barrett, 2010; Gonela et al., 2013; Guidugli et al., 2016; Souza et al., 2018; Cuénin et al., 2019; Garcia et al., 2019; Seoane et al., 2019). In highly fragmented landscapes, even low levels of gene dispersal are sufficient to counteract the long-term detrimental effects of inbreeding and loss of genetic diversity resulting from genetic drift, founder effects, and genetic erosion (e.g., Young, Boyle & Brown, 1996; Ellstrand, 2014). As an illustrative example, in the same fragmented landscape where *Adenanthera* species occurs, a gene flow study involving the allogamous species *Carianiana estrellensis* (Lecythidaceae) revealed a lack of inbreeding as a consequence of extensive gene immigration and high outcrossing rates (Guidugli et al., 2016). Therefore, to formulate biological diversity conservation policies and management programs, mainly in fragmented landscapes, an understanding of the mating system parameters (e.g., outcrossing, selfing and biparental inbreeding rates) in addition to estimates of contemporary gene flow in plant populations are indispensable (e.g., Allard, 1999; Finkeldey, 2005; Guidugli et al., 2016; Zhang et al., 2019; Lompo et al., 2020).

In this context, and considering the resilience of *Anadenanthera* species in this hyperfragmented region and their economic importance, there is considerable interest in increased knowledge of mating parameters in natural populations of *A. colubrina* and *A. peregrina*. We focused our efforts on characterizing the mating parameters for these insect-pollinated and autochorous tropical tree species. Our specific goals were to: (1) document the mating system estimates of *A. colubrina* and *A. peregrina*, (2) apply paternity analysis to quantify realized pollen dispersal distances, and (3) identify pollen immigration for *A. colubrina* and *A. peregrina*. The study of these genetic parameters in trees of the *Anadenanthera* genus in Atlantic Rainforest remnants will provide a useful starting point for conservation and management practices.

## Materials and Methods

### Study species

**Figure 1:**
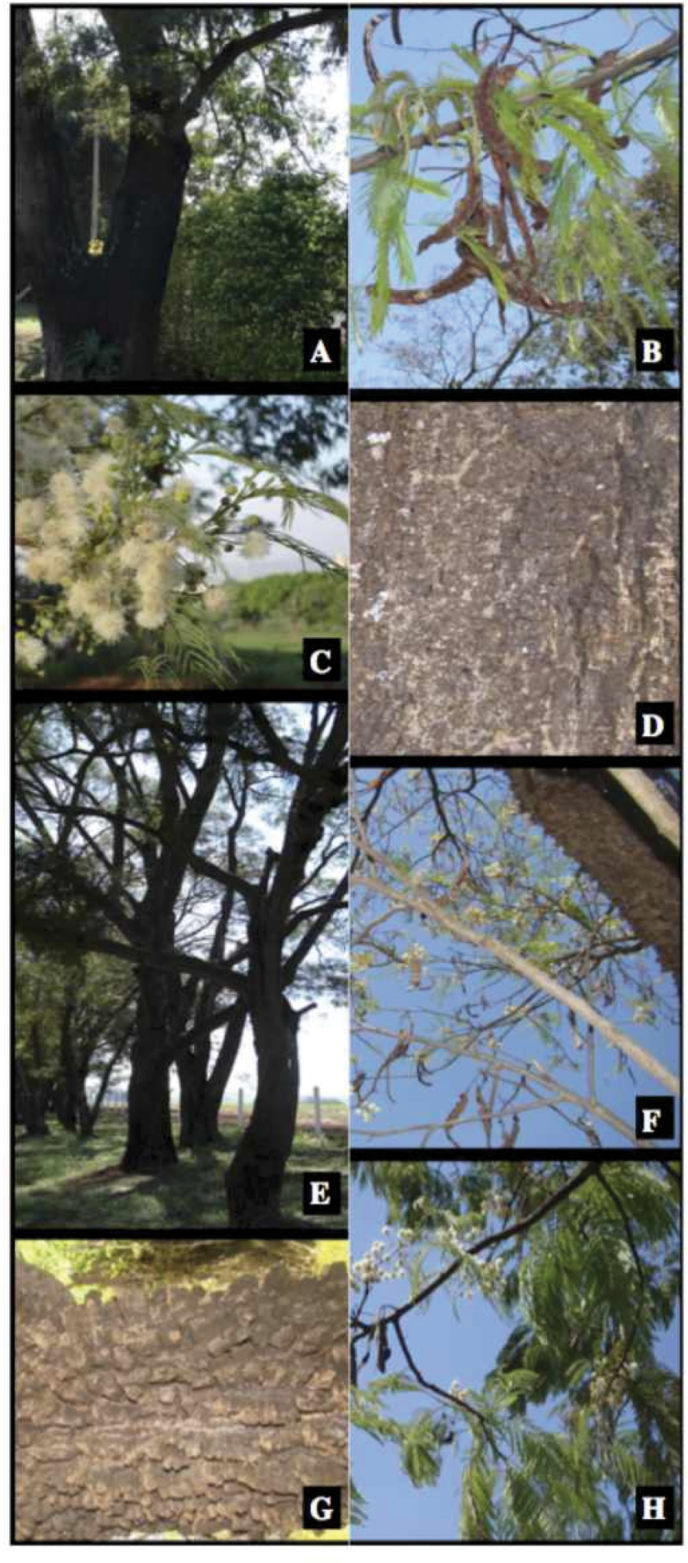
Pictures of *Anadenanthera* *colubrina* and *A*. *peregrina* illustrating its vegetative and reproductive characteristics. Highlight for the stem (A), pods (B), flowers (C), and suber (D) of *A. colubrina*. Details of the stem (E), pods (F), suber (G), and leaves and fruits (H) of *A. peregrina* are also shown.

*Anadenanthera* belongs to Fabaceae, subfamily Caesalpinioideae (LPWG, 2017). Individuals [*A. colubrina* and *A. peregrina*] are heliophyte climax trees that can reach up to 35 m in height, and a diameter at breast height (DBH) up to 120 cm. The distribution of *Anadenanthera* appears to be largely natural, except for the probable anthropogenic introduction of *A. peregrina* into the West Indies (Altschul, 1964). The species of this small genus occur in savannas (i.e., Brazilian cerrado), and in wet and dry forests throughout tropical and temperate America (Altschul, 1964). These species tolerate sandy and shallow soils and light shading during the juvenile phase. *A. colubrina* (Figs. 1A–1D) has nitid, smooth to reticulated pods, and glandular anthers, while *A. peregrina* (Figs. 1E–1H) has dull, scurfy to verrucose pods, and eglandular anthers (Altschul, 1964). *Anadenanthera colubrina* var. *colubrina* and *A. peregrina* var. *falcata*—the focal taxa of this study—are hermaphrodites with actinomorphic flowers that are pollinated by bees and small insects. In São Paulo, Brazil, both species bloom from August to October (Fig. 1). The fruits, which are pods with 8–16 seeds, ripen from August to September of the following year (Fig. 1). The seeds of both species are brown, slightly flattened, orbicular, without a wing, and range from 10 to 20 mm in diameter (Altschul, 1964) and from 0.10 g to 0.17 g in weight. Due to autochorous seed dispersal, the dry seeds are dispersed primarily over short distances (Lorenzi, 2002). Despite their economic, environmental, and cultural importance, no study has yet examined the conservation of these valuable species.

### Study site

**Figure 2:**
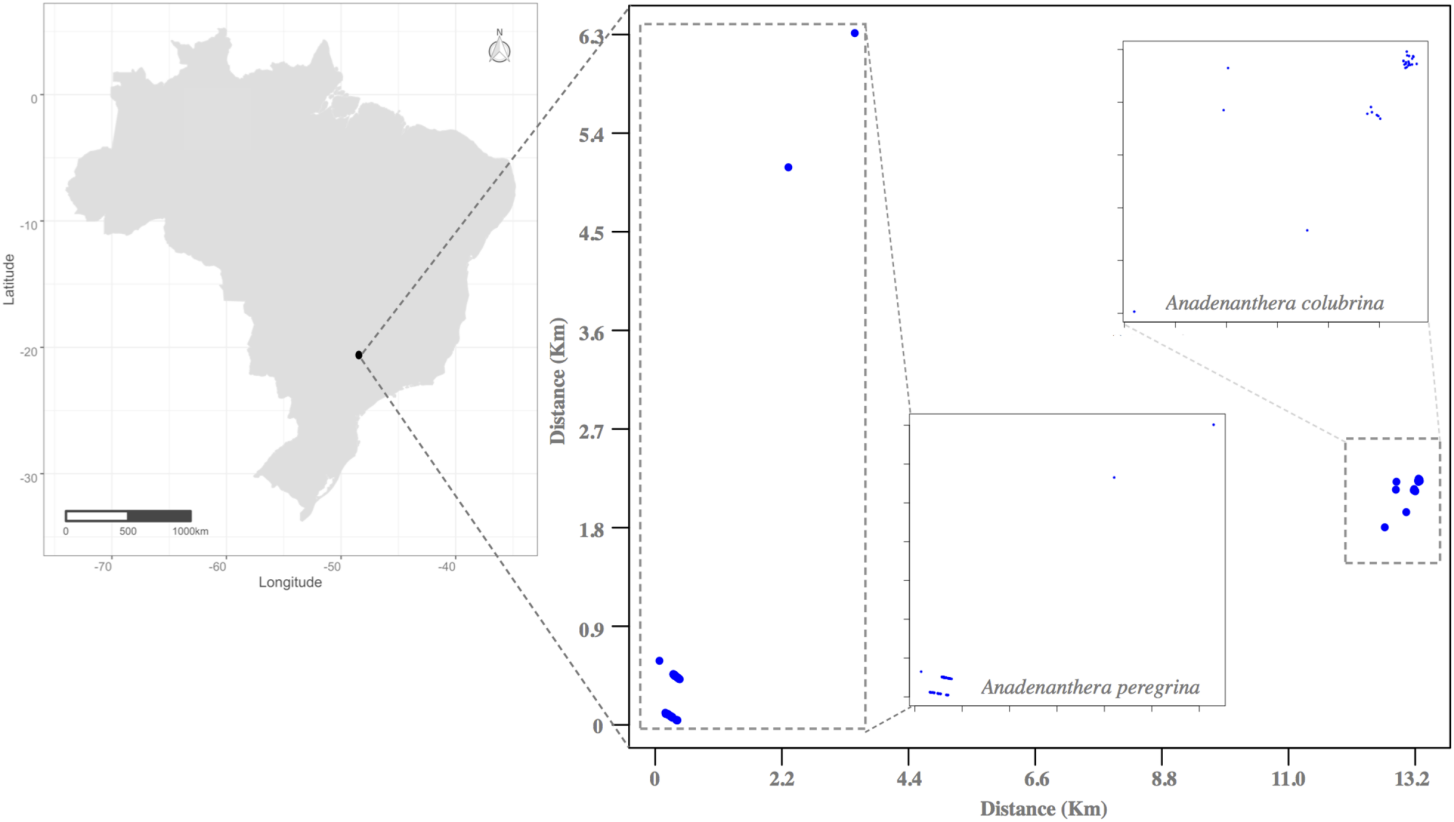
Location of the Atlantic Forest fragments studied in the municipality of Ribeirão Preto (São Paulo State, Southeastern Brazil), where individuals of the species *Anadenanthera colubrina* and *A. peregrina* were sampled.

This study was conducted in Ribeirão Preto, State of São Paulo, Southeastern Brazil, during the drought seasons of 2011 and 2012 in small, semi-deciduous forest fragments (Fig. 2). The history of forest devastation of Ribeirão Preto region began with the increase of the area for coffee production in the late nineteenth century and the massive arrival of European immigrants after the abolition of slavery in Brazil in the early twentieth century. The degradation of the few forest remnants that persist in the region continues due to the extensive production of sugarcane and intense urban expansion (Kotchetkoff-Henriques, 2003; Victor et al., 2005; Guidugli et al., 2016). Today this Region is known as one of the largest sugarcane producers in Brazil. In this context, to perform the mating system and pollen flow studies, we selected and sampled all individuals from an isolated area for each *Anadenanthera* species: Acol (*A. colubrina*, 21°17′19.86″ S, 47°48′49.29″ O, 2.7 ha, Fig. 2) and Aper (*A. peregrina*, 21°23′51.98″ S, 47°51′49.23″ O, 4.0 ha, Fig. 2). The distances among Acol and Aper adult trees were ~2.5 m to 1,300 m. In both *angicais*, we observed little regeneration, as these clusters are located along roadsides and profoundly disturbed by anthropogenic activities of urban sprawl and agriculture. Small remnants occur within 100 and 1,300 m of the sampled *angicais*, and other fragments containing the species occur between 11.0 and 20.0 km away.

### Sample collection and genetic analysis

Leaf material for *Anadenanthera colubrina* (Acol) and *A. peregrina* (Aper) was sampled from 30 and 55 adult trees, respectively, which were identified and mapped with a GPS device (Garmin, USA). The adult trees (DBH 1.4–3.9 m) were those that produced fruits during the sampling period. In addition, on average, 10 and 15 random fruits were collected from 10 adult seed-trees each of *A. colubrina* and *A. peregrina* population that flowered in 2011 and 2012, respectively. All fruits were separated, identified, and stored in paper bags (one fruit per bag) to maintain the provenance of fruit, seed, and seed-tree. The identification of seeds by fruit allowed us to determine the correlation of paternity between and within fruits. Seeds were planted in a nursery of forest seedlings of the Campus of the University of São Paulo, Ribeirão Preto. Leaf samples were obtained from 322 Acol progeny arrays (28, 33, and 30 open-pollinated progeny from one, each of eight, and one seed-trees, respectively). Likewise, we obtained leaf samples of 300 Aper progeny arrays (30 open-pollinated progeny from each seed-tree). Samples from all 85 adult trees and 622 progeny were conserved at −20 °C until DNA extraction. Research permit to collect the *Anadenanthera* species was approved by SisGen (number A380C57).

Genomic DNA extraction and microsatellite amplification conditions were described in Alzate-Marin et al. (2009) and Feres et al. (2012b), respectively. For *A. colubrina*, we scored the loci *Acol02, Acol05, Acol15, Acol16, Acol17, Acol18, Acol20*. A different set of primers (*Acol09, Acol11, Acol12, Acol13, Acol14, Acol15, Acol20*) was used for *A. peregrina* due to cross-amplification issues (Feres et al., 2012b). Electrophoretic conditions for these loci were described in Feres et al. (2012b).

### Linkage disequilibrium, Hardy-Weinberg equilibrium, and null alleles tests

The linkage disequilibrium for all the analyzed loci was tested using FSTAT 2.9.3.2 program (Goudet, 2002) applying the Bonferroni correction (Rice, 1989). Tests for deviations from Hardy-Weinberg equilibrium for adult trees and progeny arrays were examined based on Monte Carlo permutations of alleles using the adegenet package (Jombart, 2008) implemented in R 3.3.1 (R Core Team, 2018). The presence and frequency of null alleles were estimated following the method of Brookfield (1996) using the PopGenReport package (Adamack & Gruber, 2014) in R.

### Determination of mating system

Genotypes from the offspring (*n* = 322 for *A. colubrina* and *n* = 300 for *A. peregrina*) and seed-trees were evaluated using the mating system program MLTR version 3.2 (Ritland, 2008). This program took into account that all loci may contain null alleles even if there are none (Ritland, 2008). The parameters estimated for individual families (*n* = 10 families) and at the population level, included *t*_*m*_ (multilocus outcrossing rate), *t*_*s*_ (single-locus outcrossing rate), 1– *t*_m_ (selfing rate), *t*_m_–*t*_s_ (biparental inbreeding or mating among relatives), and *r*_*p(m)*_ (multilocus paternity correlation). The average number of pollen donors *N*_ep_ = 1/*r*_*p*(*m*)_ (Ritland, 1989) and the inbreeding coefficient for seed-trees and offspring were also calculated. For all parameters, the standard error (SE) was calculated from 1,000 bootstrap replicates with resampling among families.

### Categorical parentage analysis

Progeny arrays for both *Anadenanthera* species (Acol = 322, Aper = 300) were examined to estimate gene flow through pollen dispersal, considering all adult trees (*n* = 30 for *A. colubrina* and *n* = 55 for *A. peregrina*) as paternal candidates. Paternity analyses were performed according to the maximum likelihood method (Marshall et al., 1998) integrated in the CERVUS program v.3.0.3 (Kalinowski, Taper & Marshall, 2007). We carried out 50,000 simulated genotypes to achieve the critical value of Delta (Δ_crit_), considering 90% of putative fathers were sampled and a genotyping error ratio of 1%. This genotyping error was set up to minimize the effects of null alleles on paternity estimates (Dewoody, Nason & Hipkins, 2006; Kalinowski, Taper & Marshall, 2007). After Δ_crit_ was calculated, the paternity assignment was performed to achieve the value of Delta (Δ; i.e., the difference between the highest and the second highest LOD scores). Paternity analyses for *A. colubrina* and *A. peregrina* were performed using all loci and confidence level of 95%. In the paternity analyses, only the potential father with Δ > Δcrit was considered to be the true parent of the analyzed progenies. For both *Anadenathera* species, pollen dispersal distances were calculated for progeny arrays considering the geographic coordinates of the seed-tree and the assigned pollen parent within the studied population. Furthermore, pollen immigration rate (*m*_*p*_) was estimated as the percentage of genotypes not assigned to a candidate parent within the population as proposed by Smouse & Sork (2004). We also calculated the cryptic pollen flow (*C*_*gf*_; i.e., the value that expresses the proportion of genotypes assigned to a candidate father within the sampled area when the true father is located outside there) as 1 – (1 – *P*_*2*_)*^n^*, where *n* is the number of candidate fathers within the population, and *P*_*2*_ represents the combined non-exclusion probability of the second parent, when the mother parent is known (Dow & Ashley, 1996). *P*_*2*_ values were calculated from adult trees of *A. colubrina* and *A. peregrina* using the CERVUS program (Kalinowski, Taper & Marshall, 2007). Finally, considering a circular area around each seed-tree of *A. colubrina* and *A. peregrina*, we calculated the effective pollination neighborhood area (*A*_*ep*_ = 2πσ^2^) from the variance of pollen dispersal distances (σ^2^) according to Levin (1988).

## Results

### Linkage disequilibrium, Hardy-Weinberg equilibrium, and null alleles frequency

For both *A. colubrina* and *A. peregrina*, the linkage disequilibrium was not significant between loci for adults and progeny arrays after a sequential Bonferroni correction for *k* tests (*k* = 21, *p* < 0.0024). In addition, no significant departures from HWE were observed after a Bonferroni adjustment (*p* > 0.007) for adult and progeny arrays of both species. PopGenReport revealed that locus *Acol18* may contain null alleles at moderate frequencies (0.138) in adult trees of *A. colubrina* (Table S1). For *A. peregrina*, frequencies for locus *Acol20* ranged from 0.074 (adult trees) to 0.126 (progeny arrays; Table S1). However, we chose to include *Acol18* and *Acol20* in this study and accounted for null alleles in our mating system and paternity analyses.

### Mating system of *Anadenanthera* species

The average multilocus outcrossing rates estimated for *Anadenanthera colubrina* (*t*_*m*_ = 0.619) and *A. peregrina* (*t*_*m*_ = 0.905) were significantly different from unity, indicating that *A. colubrina* is a mixed mating species while *A. peregrina* is a predominantly outcrossing species with a likely absence of self-incompatibility mechanisms in both species (Table 1). Estimates of individual multilocus outcrossing rates showed variation between sampled seed-trees, with high and low observed values for the ten open-pollinated families analyzed, varying from 0.19 to 1.2 for *A. colubrina* and from 0.83 to 1.2 for *A. peregrina* (Table 1).

**Table 1:** Estimates of mating system for *Anadenanthera colubrina* (Vellozo) Brenan and *A. peregrina* (Lineau) Spegazzini sampled in populations located in Ribeirão Preto, Sao Paulo State, Southeast Brazil.

Parameters	*A. colubrina*	*A. peregrina*
Number of seed-trees/progeny arrays	10/322	10/300
Multilocus outcrossing rate (*t*_*m*_)	0.619 [0.57–0.66]	0.905 (0.89–0.92]
Multilocus outcrossing rate by family		
Family [01]	1.200 [1.02–1.37]	0.791 [0.77–0.82]
Family [02]	0.866 [0.72–1.01]	1.200 [1.19–1.21]
Family [03]	0.677 [0.57–0.79]	0.875 [0.84–0.91]
Family [04]	0.296 [0.25–0.34]	0.875 [0.84–0.91]
Family [05]	0.655 [0.55–0.76]	0.833 [0.80–0.86]
Family [06]	0.713 [0.59–0.84]	0.901 [0.87–0.93]
Family [07]	0.628 [0.52–0.74]	1.200 [1.19–1.21]
Family [08]	0.624 [0.52–0.73]	0.830 [0.80–0.86]
Family [09]	0.477 [0.40–0.56]	0.931 [0.90–0.96]
Family [10]	0.196 [0.01–0.23]	0.961 [0.92–1.00]
Single-locus outcrossing rate (*t*_*s*_)	0.460 [0.42–0.50]	0.690 [0.67–0.71]
Mating rate among relatives (*t*_*m*__-_ *t*_*s*_)	0.159 [0.12–0.19]	0.216 [0.20–0.24]
Selfing rate (1 – *t*_m_)	0.381 [0.34–0.43]	0.095 [0.08–0.11]
Multilocus paternity correlation (*r*_*p*_)	0.465 [0.41–0.52]	0.677 [0.62–0.74]
Multilocus paternity correlation within fruits	0.905 [0.84–0.97]	0.805 [0.73–0.88]
Multilocus paternity correlation among fruits	0.383 [0.33–0.44]	0.298 [0.24–0.36]
Average number of pollen donors: *N*_*ep*_	2.15	1.48
Average number of pollen donors within fruits	1.10	1.24
Average number of pollen donors among fruits	2.61	3.35
Fixation index in adult-trees	0.202*	0.091*
Fixation index in progeny arrays	0.393*	0.119*

**Notes:**

( ) 95% confidence interval obtained by 1,000 bootstraps. Confidence intervals that fall within 1 (for *t*_m_ and *t*_s_ estimates) or 0 (for *t*_m_–*t*_s_ estimate) are not significant.

* *p* < 0.05.

We detected biparental inbreeding (Ritland, 2002) for both *Anadenanthera* species in the studied populations (*t*_*m*_ − *t*_*s*_ ranged from 0.159 for *A. colubrina* to 0.216 for *A. peregrina*), indicating that 15.9% and 21.6% of crosses were realized between related individuals. The fixation index observed in the adults (*F*_*A. colubrina*_ = 0.202, *F*_*A. peregrina*_ = 0.091) and progeny arrays (*F*_*A. colubrina*_ = 0.393, *F*_*A. peregrina*_ = 0.119) of both species were statistically different from zero, indicating inbreeding (Table 1).

The multilocus paternity correlation (*r*_*p(m)*_) was high and different from zero (0.465 for *A. colubrina* and 0.677 for *A. peregrina*). However, these values were even higher when we divided the analysis into within and among fruit for each seed-tree. Within fruit, the same parents were responsible for 90.5% (*A. colubrina*) and 80.5% (*A. peregrina*) of crossings. Among fruit, we observed 38.3% (*A. colubrina*) and 29.8% (*A. peregrina*) of crossings generated by the same trees. These results indicate that the progenies are composed of a mixture of half-sibs and full-sibs. Based on paternity correlation, it was estimated that 2.15 and 1.48 effective pollen donors mated with each seed-tree in both *A. colubrina* and *A. peregrina*, respectively. However, different fruits of the same seed-tree received pollen from approximately three individuals (2.6 for *A. colubrina*, 3.35 for *A. peregrina*), whereas a single fruit may have received pollen from only one tree (Table 1).

### Paternity analysis for *Anadenanthera* species

**Figure 3:**
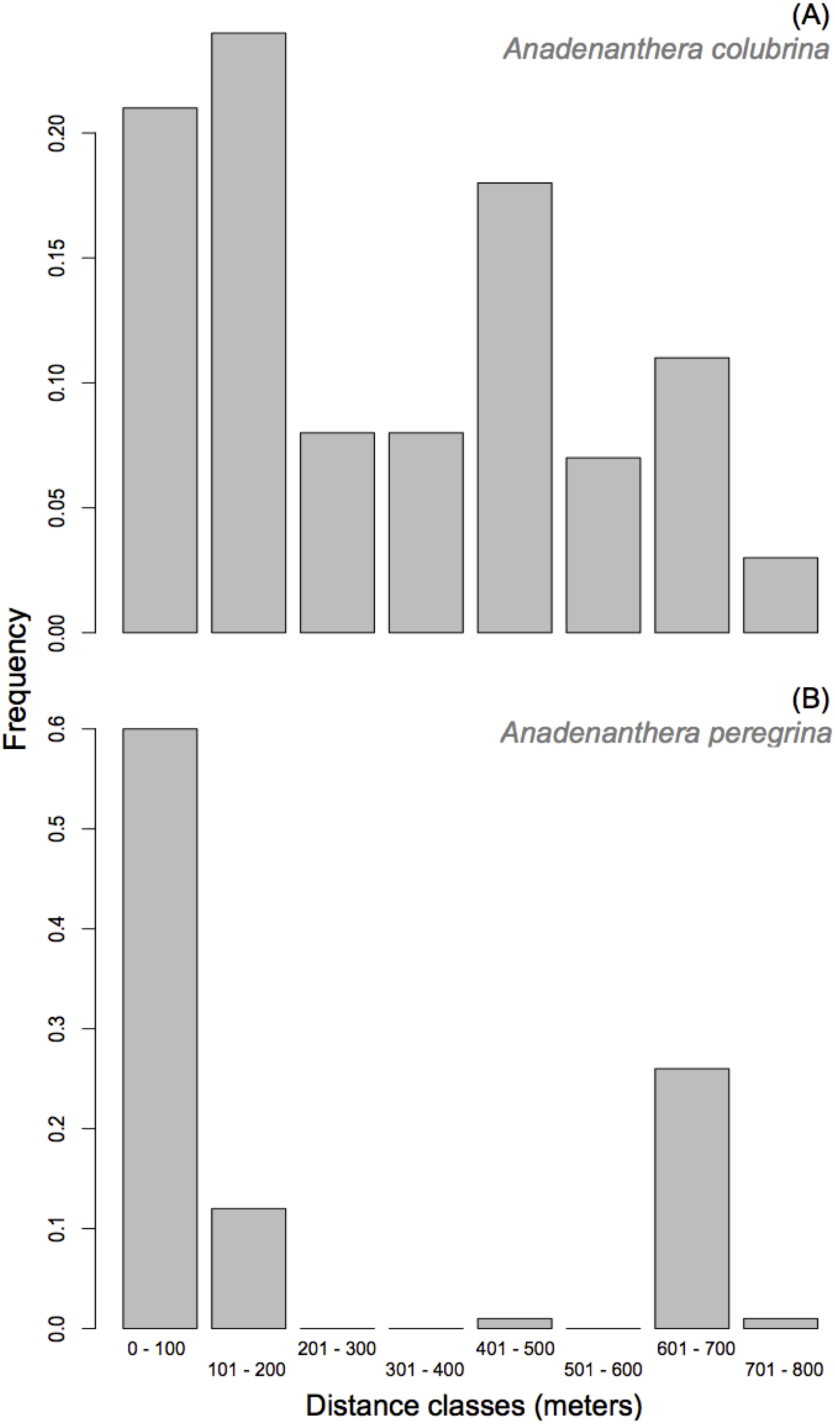
Frequency distribution of pollen dispersal distance in *Anadenanthera colubrina* (A) and *A. peregrina* (B).

*Anadenanthera colubrina*—A paternal parent was assigned to 70% of the population (225 out of 322 progeny arrays) with a minimum 80% confidence. Consistent with the results for the mating system, 103 of the 225 assigned paternities were the result of self-fertilization. Paternity was designated for 81 of the 322 progeny arrays (25%) with 95% confidence. Further, 22 of the 30 adult trees contributed paternity to offspring from the ten seed-trees analyzed, though only five donated pollen to 52% of offspring. The percentage of progenies that do not have a parent tree assigned within the studied population (i.e., pollen immigration) was moderate (*m_p_* = 30.0%). Since the cryptic gene flow had a probability of 6.3%, the total pollen migration for progeny arrays was 36%, which indicates that this small *A. colubrina* population is not genetically isolated given the exclusion probability (*P*_*2*_ = 0.919; see Table S2) and sampling of all adult trees within the population. Effective pollen dispersal distances within the population were relatively short, with 53% of pollination occurrences at distances up to 300 m (Fig. 3). However, 39% of the effective pollination was observed over long distances (>500 m; Fig. 3). Overall, the pollen dispersal distance ranged from 7 to 769 m, with an average of 299.8 ± 8.4 m (SE). The effective pollination neighborhood (*A*_ep_) for *A. colubrina* ranged from 0.57 to 5.73 ha, with an average of 2.54 ha between seed-trees, corresponding to a circle around a seed-tree with a radius of 90 m.

*Anadenanthera peregrina*—For this species, a paternal parent was assigned to 64% of the population (193 of 300 progeny arrays) with a minimum 80% confidence. Paternity was designated with 95% confidence for 60 of the 300 sampled progeny arrays (20%). Further, 45 of the 55 adult trees contributed pollen to offspring from the analyzed 10 seed-trees, and only 11 pollinated 50% of offspring. The percentage of pollen immigration was moderate (*m*_p_ = 35.0%). Since the cryptic gene flow had a probability of 5.4%, the total pollen immigration for progeny arrays was 40.4%, indicating that the forest fragment is not genetically isolated, given the exclusion probability (*P*_*2*_ = 0.970; see Table S2) and sampling of all adult trees within the population, a result similar to *A. colubrina*. Although 27% of the effective pollination was observed over long distances (>700 m; Fig. 3), 72% of dispersal distances within the population were relatively short (i.e., up to 200 m; Fig. 3). The effective pollination neighborhood (*A*_ep_) ranged from 0.54 to 307.3 ha, with an average of 17.16 ha between seed-trees, corresponding to a circle around a seed-tree with a radius of 234 m.

## Discussion

To our knowledge, this is the first study assessing the mating system and gene flow patterns of the economically and ecologically important leguminous trees *A. colubrina* and *A. peregrina*. The mating system was studied in two small natural clusters (*angicais*) of these species located in Ribeirão Preto, Brazil, an area in which the forest has been highly fragmented for at least 100 years. Both species of *Anadenanthera* presented a mixed-mating system, with selfing, mating between relatives, and a limited number of pollen donors. However, *A. peregrina* presents a higher outcrossing rate than *A. colubrina*. These estimates are comparable to those reported in field experiments in another region of Brazil (Mato Grosso State), where *A. peregrina* is identified as an allogamous species with a self-pollination rate of 9.48% (Costa, Contini & Melo, 2003). Protandry is also reported for both *Anadenanthera* species as one of the cross-pollination strategies for the species (Costa, Kageyama & Mariano, 1992; Costa, Contini & Melo, 2003). However, to confirm that mixed-mating is indeed a specific characteristic of these species, more populations in other regions and biomes in Brazil must be analyzed.

The estimates for reproductive parameters for both *Anadenanthera* species show a wide variety among the sampled seed-trees, which may indicate that the selfing rate fluctuates between reproductive events. Furthermore, our results suggest that the reproductive system of *A. colubrina* and *A. peregrina* may not be resilient to reductions in population size, since the results for biparental inbreeding indicate mating among relatives. Although we have not investigated the spatial genetic structure in these populations due to the small sample sizes mainly for *A. colubrina*, it can be inferred based on the occurrence of pollen flow over relatively short distances and the biparental inbreeding observed in the studied populations. These results are consistent with other tree species in fragmented populations that have shown elevated levels of mating between related individuals (Ismail et al., 2012; Tambarussi et al., 2017; Souza et al., 2018) and inbreeding (Fuchs, Lobo & Quesada, 2003; Jump & Peñuelas, 2006; Kettle et al., 2007; Dick et al., 2008; Vranckx et al., 2011; Zhang et al., 2012; Finger et al., 2014; Tambarussi et al., 2017; Seoane et al., 2019; but see Lompo et al., 2020). Such findings could be explained by the genetic structure of natural clusters (i.e., *angicais*) in the parental generation, with near-neighbor related trees resulting from seed dispersal close to the seed-tree (autochoric dispersion), profuse flowering, and pollinator behavior. In the present study, the pollination biology of *Anadenathera* species was not investigated; however, other authors have reported that inflorescence of *Anadenanthera* species are foraged intensively by native bees, including *Trigona spinipes* (Fabr.), *Plebeia sp*., *Frieseomelitta sp*., and the exotic bee *Apis mellifera* (Teixeira, Oliveira & Viana, 2007; Trovão et al., 2009). Therefore, the population structure of reproductive adult trees in the fragmented habitat and pollinator behavior are key aspects in the crossing rates observed herein and may play an essential role in connecting the few remaining forest fragments.

Gene flow by pollen dispersal outside the boundaries of forest fragments has been reported for several animal-pollinated plant species (Nason & Hamrick, 1997; Sato et al., 2006; Nazareno & Carvalho, 2009; Ottewell et al., 2012; Côrtes et al., 2013; Saro et al., 2014; Guidugli et al., 2016; Garcia et al., 2019; Skogen et al., 2019; Lompo et al., 2020). Even in an extremely fragmented landscape, such as the Ribeirão Preto region, we observed a moderate frequency (30–35%) of pollen immigration for both *Anadenanthera* species, showing that pollen flow outside the edges of small forest fragments is feasible. In this same region, Guidugli et al. (2016) also reported extensive pollen immigration (i.e., up to 53%) for the allogamous tree species *Cariniana estrellensis* in a forest fragment size of 8.0 ha. The main point raised by these two studies, and with potential relevance for conservation strategies in Ribeirão Preto region, is that populations of tree species in small forest fragments are not genetically isolated.

In practical terms, our study presents basic information that should be considered for in situ strategies, and when collecting seeds for breeding, conservation, and restoration programs. For seed collection, it should be considered that all seeds in a fruit of *A. colubrina* and *A. peregrina* are a mixture of half- and full-sibs, and that seed-trees of both species were pollinated by multiple pollen donors. Hence, we recommend collecting one seed from many separate fruits through the whole crown of each seed-tree. However, further fine-scale spatial genetic structure studies should be documented for these populations to avoid collecting seeds from genetically related seed-trees. With regards to gene flow, although the estimates are biased due to the low power of exclusion of the seven loci used herein, we can conclude that the *Anadenanthera* populations received a moderate percentage of immigrant pollen. Although pollen dispersal occurred predominantly over relatively short distances in the *A. colubrina* and *A. peregrina* populations, pollen flow over long distances was also observed (Fig. 3). These dispersal patterns are relevant for the in situ conservation of remaining local populations since gene flow over long distances can enhance and/or increase connectivity between the remaining fragments of *Anadenanthera* species. Indeed, this suggests that *angicais* could play the role of ecological stepping stones while also enabling the viability of local pollinators. Several empirical studies have shown the importance of connectivity for processes of recolonization after local extinction, as well as in the maintenance of metapopulations across fragmented landscapes (e.g., Hanski, 1994; Metzger, 2000; Antongiovanni & Metzger, 2005; Rubio & Saura, 2012; Laurance et al., 2018), as appears to be the case with *angicais* in the Ribeirão Preto region.

The concept of connectivity is essential, since species survive in fragmented habitats (or are dispersed heterogeneously throughout the landscape) and depend on their natural capacity for gene flow across landscape matrices. Generally, the matrix acts as a barrier to the movement of seed and pollen dispersal, restricting local populations (e.g., Metzger, 2000; Dick, Etchelecu & Austerlitz, 2003; Antongiovanni & Metzger, 2005; Uezu, Metzger & Vielliard, 2005; Ayram et al., 2016). The intensity of gene flow between habitats is regulated by the permeability of the matrix and the inherent ability of each species for pollen and seed flow across these environments (e.g., Metzger, 2000; Uezu, Beyer & Metzger, 2008). Therefore, considering the importance of connectivity between fragments to maintain forest remnant viability and biodiversity, efforts have been made to reduce habitat isolation. Several strategies have proven to increase connectivity between fragments, notably the establishment of corridors along riparian forests (e.g., Rosot et al., 2018), increasing the porosity of the matrix (e.g., Viana & Pinheiro, 1998; Rodrigues et al., 2009; Rubio & Saura, 2012). Such measures must be based on empirical data, especially related to the flow capacity of natural species particular to each environment (e.g., McRae et al., 2012; Torrubia et al., 2014). As highlighted by Ribeiro et al. (2009), small remaining forest patches (less than 50 ha) play an important role in connectivity in the fragmented landscape, reducing the average isolation among forest populations from 3,532 m to 1,441 m.

## Conclusions

Based on the study of gene flow and the mating system of *Anadenanthera* species in Ribeirão Preto region, we suggest that *angicais* play an important role connecting the few remaining local fragments. Thus, strategies for the conservation and maintenance of diversity for *angicais* and other species should be based on the information reported herein. Nevertheless, further studies assessing mating parameters on a spatial-temporal scale are necessary to strengthen our understanding of the mating systems and gene dispersal patterns of these tropical plant species.

## Supplemental Information

10.7717/peerj.10579/supp-1 Supplemental Information 1 Microsatellite data for *Anadenanthera* colubrina and *Anadenanthera* peregrina. Each two columns represents a specific locus. Individuals (adults and progenies) are listed in each row.

10.7717/peerj.10579/supp-2 Supplemental Information 2 The cumulative exclusion probabilities for the first (*P_1_*) and second (*P_2_*) parents for *Anadenanthera colubrina* (Vellozo) Brenan and *A. peregrina* (Lineau) Spegazzini.

10.7717/peerj.10579/supp-3 Supplemental Information 3 Null alleles frequency in *Anadenanthera* microsatellite loci, as estimated by the R package PopGenReport in adult trees and progeny arrays.

## Additional Information and Declarations

### Field Study Permissions

The following information was supplied relating to field study approvals (i.e., approving body and any reference numbers): Research permit (number A380C57) was approved by SISGEN (Sistema de Gestão do Patrimônio Genético e do Conhecimento Tradicional Associado).

### Data Availability

The following information was supplied regarding data availability: Microsatellite data for *A. colubrina* and *P. peregrina* are available at Dryad (DOI 10.5061/dryad.1vhhmgqqg).

## References

[ref-1] Adamack AT, Gruber B (2014). PopGenReport: simplifying basic population genetic analyses in R. Methods in Ecology and Evolution.

[ref-2] Allard RW (1999). Principles of plant breeding.

[ref-48] Altschul SVR (1964). A taxonomic study of the genus *Anadenanthera*. Contributions from the Gray Herbarium of Harvard University.

[ref-3] Alzate-Marin AL, Guidugli MC, Soriani HH, Martinez CAH, Mestriner MA (2009). A DNA minipreparation procedure suitable for PCR/SSR and RAPD analyses in tropical forest tree species. Brazilian Archives of Biology and Technology.

[ref-4] Antongiovanni M, Metzger JP (2005). Influence of matrix habitats on the occurrence of insectivorous bird species in Amazonian forest fragments. Biological Conservation.

[ref-5] Ayram CAC, Mendoza ME, Etter A, Salicrup DRP (2016). Habitat connectivity in biodiversity conservation: a review of recent studies and applications. Progress in Physical Geography.

[ref-6] Barrett SCH (2010). Understanding plant reproductive diversity. Philosophical Transactions of the Royal Society B: Biological Sciences.

[ref-7] Brookfield JFY (1996). A simple new method for estimating null allele frequency from heterozygote deficiency. Molecular Ecology.

[ref-8] Carvalho PER (2003). Espécies arbóreas brasileiras.

[ref-9] Côrtes MC, Uriarte M, Lemes MR, Gribel R, Kress WJ, Smouse PE, Bruna EM (2013). Low plant density enhances gene dispersal in the Amazonian understory herb *Heliconia acuminata*. Molecular Ecology.

[ref-10] Costa RB, Contini AZ, Melo ESP (2003). Reproductive system of *Anadenanthera peregrina* and *Vochysia haenkiana* in a fragment of “Cerrado forest” from Chapada dos Guimarães—MT. Brazil Ciência Rural.

[ref-11] Costa RB, Kageyama PY, Mariano G (1992). Breeding system study of Anadenanthera falcata Benth., Vochysia tucanorum Mart. and Xylopia aromatica Baill., in a Cerrado area. Revista Brasileira de Sementes.

[ref-12] Cuénin N, Flores O, Rivière E, Lebreton G, Reynaud B, Martos F (2019). Great genetic diversity but high selfing rates and short-distance gene flow characterize populations of a tree (*Foetidia*; Lecythidaceae) in the fragmented tropical dry forest of the Mascarene Islands. Journal of Heredity.

[ref-13] Dick CW, Etchelecu G, Austerlitz F (2003). Pollen dispersal of Neotropical trees (*Dinizia excelsa*: Fabaceae) by native insects and African honeybees in pristine and fragmented Amazonian rainforest. Molecular Ecology.

[ref-14] Dick CW, Hardy OJ, Jones FA, Petit RJ (2008). Spatial scales of pollen and seed-mediated gene flow in tropical rain forest trees. Tropical Plant Biology.

[ref-15] Dewoody J, Nason JD, Hipkins VD (2006). Mitigating scoring errors in microsatellite data from wild populations. Molecular Ecology Notes.

[ref-16] Dow BD, Ashley MV (1996). Microsatellite analysis of seed dispersal and parentage of sampling in bur oak, *Quercus macrocarpa*. Molecular Ecology.

[ref-17] Durigan G, Silveira ER (1999). Riparian forest restoration in cerrado, Assis, SP. Brazil Scientia Forestalis.

[ref-18] Ellstrand NC (2014). Is gene flow the most important evolutionary force in plants?. American Journal of Botany.

[ref-19] Feres JM, Guidugli MC, Mestriner MA, Sebbenn AM, Ciampi AY, Alzate-Marin AL (2009). Microsatellite diversity and effective population size in a germplasm bank of *Hymenaea courbaril* L. var stilbocarpa (Leguminosae), an endangered tropical tree: recommendations for conservation. Genetic Resources and Crop Evolution.

[ref-20] Feres JM, Sebbenn AM, Guidugli MC, Mestriner MA, Moraes MLT, Alzate-Marin AL (2012a). Mating system parameters at hierarchical levels of fruits, individuals and populations in the Brazilian insect-pollinated tropical tree, *Tabebuia roseo-alba* (Bignoniaceae). Conservation Genetics.

[ref-21] Feres JM, Monteiro M, Zucchi MI, Pinheiro JB, Mestriner MA, Alzate-Marin AL (2012b). Development of microsatellite markers for *Anadenanthera colubrina* (Leguminosae), a neotropical tree species. American Journal of Botany.

[ref-22] Finger A, Kaiser-Bunbury CN, Kettle CJ, Valentin T, Ghazoul J (2014). Genetic connectivity of the moth pollinated tree *Glionnetia sericea* in a highly fragmented habitat. PLOS ONE.

[ref-23] Finkeldey R (2005). An introduction to tropical forest genetics.

[ref-24] Fuchs EJ, Lobo JA, Quesada M (2003). Effects of forest fragmentation and flowering phenology on the reproductive success and mating patterns of the tropical dry forest tree *Pachira quinata*. Conservation Biology.

[ref-25] Garcia AS, Bressan EA, Ballester MVR, Figueira A, Sebbenn AM (2019). High rates of pollen and seed flow in *Hymenaeae stignocarpa* on a highly fragmented savanna landscape in Brazil. New Forests.

[ref-26] Gonela A, Sebbenn AM, Soriani HH, Mestriner MA, Martinz CAM, Alzate-Marin AL (2013). Genetic diversity and mating system of *Copaifera langsdorffii* (Leguminosae, Caesalpinioideae). Genetics and Molecular Research.

[ref-27] Goudet J (2002). FSTAT: a program to estimate and test gene diversities and fixation indices (version 2.9.3) Institute of Ecology, Lausanne, Switzerland. http://www2.unil.ch/popgen/softwares/fstat.htm.

[ref-28] Guidugli M, Nazareno AG, Feres JM, Contel E, Mestriner M, Alzate-Marin AL (2016). Small but not isolated: a population genetic survey of the tropical tree *Cariniana estrellensis* (Lecythidaceae) in a highly fragmented habitat. Heredity.

[ref-29] Hanski I (1994). A practical model of metapopulation dynamics. Journal of Animal Ecology.

[ref-30] Ismail SA, Ghazoul J, Ravikanth G, Shaanker RU, Kushalappa G, Kettle CJ (2012). Does long-distance pollen dispersal preclude inbreeding in tropical trees? Fragmentation genetics of *Dysoxylum malabaricum* in an agro-forest landscape. Molecular Ecology.

[ref-31] Jombart T (2008). Adegenet: a R package for the multivariate analysis of genetic markers. Bioinformatics.

[ref-32] Jump AS, Peñuelas J (2006). Genetic effects of chronic habitat fragmentation in a wind pollinated tree. Proceedings of the National Academy of Sciences.

[ref-33] Kalinowski ST, Taper ML, Marshall TC (2007). Revising how the computer program CERVUS accommodates genotyping error increases success in paternity assignment. Molecular Ecology.

[ref-34] Kettle CJ, Hollingsworth PM, Jaffré T, Moran B, Ennos RA (2007). Identifying the early genetic consequences of habitat degradation in a highly threatened tropical conifer, *Araucaria nemorosa* Laubenfels. Molecular Ecology.

[ref-35] Kotchetkoff-Henriques O (2003). Characterization of the natural vegetation in Ribeirão Preto, SP: Bases for conservation.

[ref-36] Laurance WF, Camargo JLC, Fearnside PM, Lovejoy TE, Williamson GB, Mesquita RCG, Meyer CFJ, Bobrowiec PED, Laurance SGW (2018). An Amazonian rainforest and its fragments as a laboratory of global change. Biological Reviews.

[ref-37] Lompo D, Vicenti B, Konrad H, Duminil J, Geburek T (2020). Fine-scale spatial genetic structure, mating, and gene dispersal patterns in *Parkia biglobosa* populations with different levels of habitat fragmentation. American Journal of Botany.

[ref-38] Lorenzi H (2002). Árvores Brasileiras—manual de identificação e cultivo de plantas arbóreas nativas no Brasil. Instituto Plantarum, Nova Odessa.

[ref-39] Levin DA (1988). The paternity pool plants. American Naturalist.

[ref-78] LPWG (2017). A new subfamily classification of the Leguminosae based on a taxonomically comprehensive phylogeny. Taxon.

[ref-40] Marshall TC, Slate J, Kruuk LEB, Pemberton JM (1998). Statistical confidence for likelihood-based paternity inference in natural populations. Molecular Ecology.

[ref-41] McRae BH, Hall SA, Beier P, Theobald DM (2012). Where to restore ecological connectivity? Detecting barriers and quantifying restoration benefits. PLOS ONE.

[ref-42] Metzger JP (2000). Tree functional group richness and landscape structure in a Brazilian tropical fragmented landscape. Ecological Applications.

[ref-43] Monteiro MJ, Almeida FCBRC, Albuquerque PU, Lucena RFP, Florentino ATN, Oliveira RLC (2006). Use and traditional management of *Anadenanthera colubrina* (Vell.) Brenan in the semi-arid region of northeastern Brazil. Journal of Ethnobiology and Ethnomedicine.

[ref-44] Nason JD, Hamrick JL (1997). Reproductive and genetic consequences of forest fragmentation: two case studies of Neotropical canopy trees. Journal of Heredity.

[ref-45] Nazareno AG, Carvalho D (2009). What the reasons for no inbreeding and high genetic diversity of the Neotropical fig tree *Ficus arpazusa*?. Conservation Genetics.

[ref-46] Ottewell K, Grey E, Castillo F, Karubian J (2012). The pollen dispersal kernel and mating system of an insect-pollinated tropical palm, *Oenocarpus bataua*. Heredity.

[ref-47] R Core Team (2018). R: a language and environment for statistical computing.

[ref-49] Ribeiro MC, Metzger JP, Martensen AC, Ponzoni FJ, Hirota MM (2009). The Brazilian Atlantic Forest: how much is left, and how is the remaining forest distributed? Implications for conservation. Biological Conservation.

[ref-50] Rice WR (1989). Analyzing tables of statistical tests. Evolution.

[ref-51] Ritland K (1989). Correlated matings in the partial selfer, *Mimulus guttatus*. Evolution.

[ref-79] Ritland K (2002). Extensions of models for the estimation of mating systems using n independent loci. Heredity.

[ref-52] Ritland K (2008). Multilocus mating system program—MLTR version 3.2. University of British Columbia, Vancouver. http://genetics.forestry.ubc.ca/ritland/programs.htlm.

[ref-53] Rodrigues RR, Lima RAF, Gandolfi S, Nave AG (2009). On the restoration of high diversity forests: 30 years of experience in the Brazilian Atlantic Forest. Biological Conservation.

[ref-54] Rosot MAD, Maran JC, Luz NB, Garrastazú MC, Oliveira YMM, Franciscon L, Clerici N, Vogt P, Freitas JV (2018). Riparian forest corridors: a prioritization analysis to the landscape sample units of the Brazilian National Forest Inventory. Ecological Indicators.

[ref-55] Rubio L, Saura S (2012). Assessing the importance of individual habitat fragments as irreplaceable connecting elements: an analysis of simulated and real landscape data. Ecological Complexity.

[ref-56] Sato T, Isagi Y, Sakio H, Osumi K, Goto S (2006). Effect of gene flow on spatial genetic structure in the riparian canopy tree *Cercidiphyllum japonicum* revealed by microsatellite analysis. Heredity.

[ref-57] Saro I, Robledo-Arnuncio JJ, Gonzáles-Pérez MA, Sosa PA (2014). Patterns of pollen dispersal in a small population of the Canarian endemic palm (*Phoenix canariensis*). Heredity.

[ref-58] Seoane C, Diaz V, Kageyama PY, Moreno M, Tambarussi E, Aguiar A, Sebbenn A (2019). The Neotropical tree Ilex paraguariensis A, (Aquafoliaceae): pollen and seed dispersal in a fragmented landscape. Annals of Forest Resources.

[ref-59] Silva RMG, Saraiva TS, Silva RB, Gonçalves LA, Silva LP (2010). Allelopathy potential of etanolic extract of *Anadenanthera macrocarpa* and *Astronium graveolens*. Bioscience Journal.

[ref-60] Skogen KA, Overson RP, Hilpman ET, Fant JB (2019). Hawkmoth pollination facilitates long-distance pollen dispersal and reduces isolation across a gradient of land-use change. Annals of the Missouri Botanical Garden.

[ref-61] Smouse PE, Sork VL (2004). Measuring pollen flow in forest trees: an exposition of alternative approaches. Forest Ecology and Management.

[ref-62] Souza DL, Rodrigues E, Pinto A, Caldas MS (2007). The bees agents pollinizer’s. Revista electrónica de Veterinaria.

[ref-63] Souza FB, Kubota TYK, Tambarussi EV, Freitas MLM, Moraes MLT, Silva AM, Cambuim J, Sebbenn AM (2018). Historic pollen and seed dispersal in fragmented populations of two largest trees of the Atlantic forest. Forestry Research and Engineering: International Journal.

[ref-64] Tambarussi EV, Boshier D, Vencovsky R, Freitas MLM, Sebbenn AM (2017). Inbreeding depression from selfing and mating between relatives in the Neotropical tree Cariniana legalis Mart. Kuntze. Conservation Genetics.

[ref-65] Teixeira AR, Oliveira FF, Viana BF (2007). Utilization of floral resources by bees of the genus *Frieseomelitta* von Ihering (Hymenoptera: Apidae). Neotropical Entomology.

[ref-66] Torres CM, Repke DB (2012). Anadenanthera visionary plant in ancient South America.

[ref-67] Torrubia S, McRae BH, Lawler JJ, Hall SA, Halabisky M, Langdon J, Case M (2014). Getting the most connectivity per conservation dollar. Frontiers in Ecology and the Environment.

[ref-68] Trovão DMBM, Cruz de Souza B, Carvalho ECD, Oliveira LA, Ferreira LMR (2009). Vegetable species of the Caatinga associated the communities of bees (Hymenoptera: Apoidea: Apiformis). Revista Caatinga.

[ref-69] Uezu A, Metzger JP, Vielliard JME (2005). Effects of structural and functional connectivity and patch size on the abundance of seven Atlantic Forest bird species. Biological Conservation.

[ref-70] Uezu A, Beyer DD, Metzger JP (2008). Can agroforest woodlots work as stepping stones for birds in the Atlantic Forest region?. Biodiversity and Conservation.

[ref-71] Viana VM, Pinheiro LAFV (1998). Conservação da biodiversidade em fragmentos florestais. IPEF.

[ref-72] Victor MAM, Cavalli AC, Guillaumon JR, Serra Filho R (2005). Cem anos de devastação: revisitada 30 anos depois.

[ref-73] Vranckx GUY, Jacquemyn H, Muys B, Honnay O (2011). Meta-analysis of susceptibility of woody plants to loss of genetic diversity through habitat fragmentation. Conservation Biology.

[ref-74] Weber CR, Soares CML, Lopes ABD, Silva TS, Nascimento MS, Ximenes ECPA (2011). *Anadenanthera colubrina*: a therapeutic potential study. Revista Brasileira de Farmácia.

[ref-75] Young A, Boyle T, Brown AHD (1996). The population genetic consequences of habitat fragmentation for plants. Trends Ecology and Evolution.

[ref-76] Zhang Z, Shi M-M, Shen D-W, Chen X-Y (2012). Habitat loss other than fragmentation per se decreased nuclear and chloroplast genetic diversity in a monoecious tree. PLOS ONE.

[ref-77] Zhang Z, Gale SW, Li JH, Fischer GA, Ren MX, Song XQ (2019). Pollen-mediated gene flow ensures connectivity aming spatially discrete subpopulations of *Phalaenopsis pulcherrima*, a tropical food-deceptive orchid. BMC Plant Biology.

