# Supplementary material for: Depicting the mating system and patterns of contemporary pollen flow in trees of the genus Anadenanthera (Fabaceae)
Source: PeerJ. 2021 Apr 7;9:e10579. doi: 10.7717/peerj.10579 (PMC8035892; doi:10.7717/peerj.10579)
Supplement: Supplemental Information 2 [file peerj-09-10579-s002.docx]

**TABLE S1** Null alleles frequency in *Anadenanthera* microsatellite loci, as estimated by the R package PopGenReport in adult trees and progeny arrays.

|  | *A. colubrina* | | *A. peregrina* | |
| --- | --- | --- | --- | --- |
| **Loci** | **Adult trees** | **Progeny arrays** | **Adult trees** | **Progeny arrays** |
| Acol 02 | 0.069 | 0.053 | - | - |
| Acol 05 | -0.044 | 0.059 | - | - |
| Acol 09 | - | - | -0.014 | -0.014 |
| Acol 11 | - | - | 0.037 | 0.037 |
| Acol 12 | - | - | -0.056 | -0.056 |
| Acol 13 | - | - | 0.018 | 0.018 |
| Acol 14 | - | - | 0.039 | 0.049 |
| Acol 15 | 0.018 | 0.052 | 0.022 | 0.022 |
| Acol 16 | 0.053 | 0.049 | - | - |
| Acol 17 | 0.042 | 0.039 | - | - |
| Acol 18 | 0.138 | 0.008 | - | - |
| Acol 20 | 0.068 | 0.027 | 0.074 | 0.126 |
