# Supplementary material for: Depicting the mating system and patterns of contemporary pollen flow in trees of the genus Anadenanthera (Fabaceae)
Source: PeerJ. 2021 Apr 7;9:e10579. doi: 10.7717/peerj.10579 (PMC8035892; doi:10.7717/peerj.10579)
Supplement: Supplemental Information 3 [file peerj-09-10579-s003.docx]

**TABLE S2** The cumulative exclusion probabilities for the first (*P_1_*) and second (*P_2_*) parents for *Anadenanthera colubrina* (Vellozo) Brenan and *A. peregrina* (Lineau) Spegazzini.

|  | *A. colubrina* | | *A. peregrina* | |
| --- | --- | --- | --- | --- |
| **Loci** | ***P_1_*** | ***P_2_*** | ***P_1_*** | ***P_2_*** |
| Acol 02 | 0.882 | 0.726 | - | - |
| Acol 05 | 0.974 | 0.898 | - | - |
| Acol 09 | - | - | 0.930 | 0.804 |
| Acol 11 | - | - | 0.730 | 0.567 |
| Acol 12 | - | - | 0.742 | 0.581 |
| Acol 13 | - | - | 0.809 | 0.672 |
| Acol 14 | - | - | 0.605 | 0.428 |
| Acol 15 | 0.659 | 0.476 | 0.772 | 0.590 |
| Acol 16 | 0.827 | 0.664 | - | - |
| Acol 17 | 0.633 | 0.454 | - | - |
| Acol 18 | 0.991 | 0.932 | - | - |
| Acol 20 | 0.990 | 0.931 | 0.807 | 0.664 |
| **Cumulative** | **0.709** | **0.919** | **0.846** | **0.970** |
